# Supplementary material for: Complete Cycle Mapping Using a Quantitative At-Home Hormone Monitoring System in Prediction of Fertile Days, Confirmation of Ovulation, and Screening for Ovulation Issues Preventing Conception
Source: Medicina (Kaunas). 2022 Dec 15;58(12):1853. doi: 10.3390/medicina58121853 (PMC9783738; doi:10.3390/medicina58121853)
Supplement: Supplementary file 1 [file medicina-58-01853-s001.zip › medicina-2013387-supplementary.pdf]

Complete cycle mapping using a quantitative at-home hormone monitoring system in prediction of fertile days, confirmation of ovulation, and screening for ovulation issues preventing conception

Andrea K. Wegrzynowicz[1,2], Amy Beckley*[1], Aimee Eyvazzadeh[3], Gary Levy[4], John Park[5], Joshua Klein[6]

Supplemental Materials

Table of Contents:
Figure S1: Proov Insight App algorithmically calculates hormone values
Figure S2: Quality control is based on best-fit line parameters
Table S1: Multihormone strips show no cross-reactivity to tested compounds
Table S2: Diagnoses and Treatments

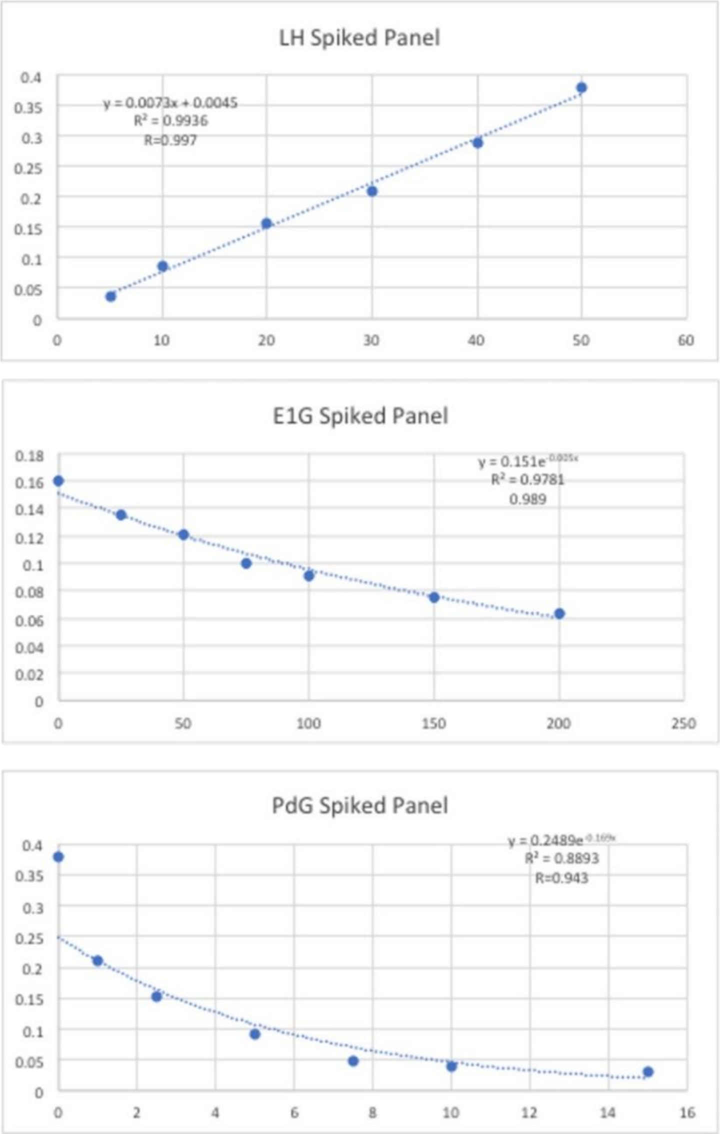

Figure S1. Quality control is based on best-fit line parameters. To ensure reproducibility of test strips, test panels for each hormone were run with sample strips from multiple lots. Sample panels and results are shown here for each hormone. Best-fit lines were created for each set of results, and reproducibility standards were met if thresholds for minimum R-values were met for 90% of strips. .

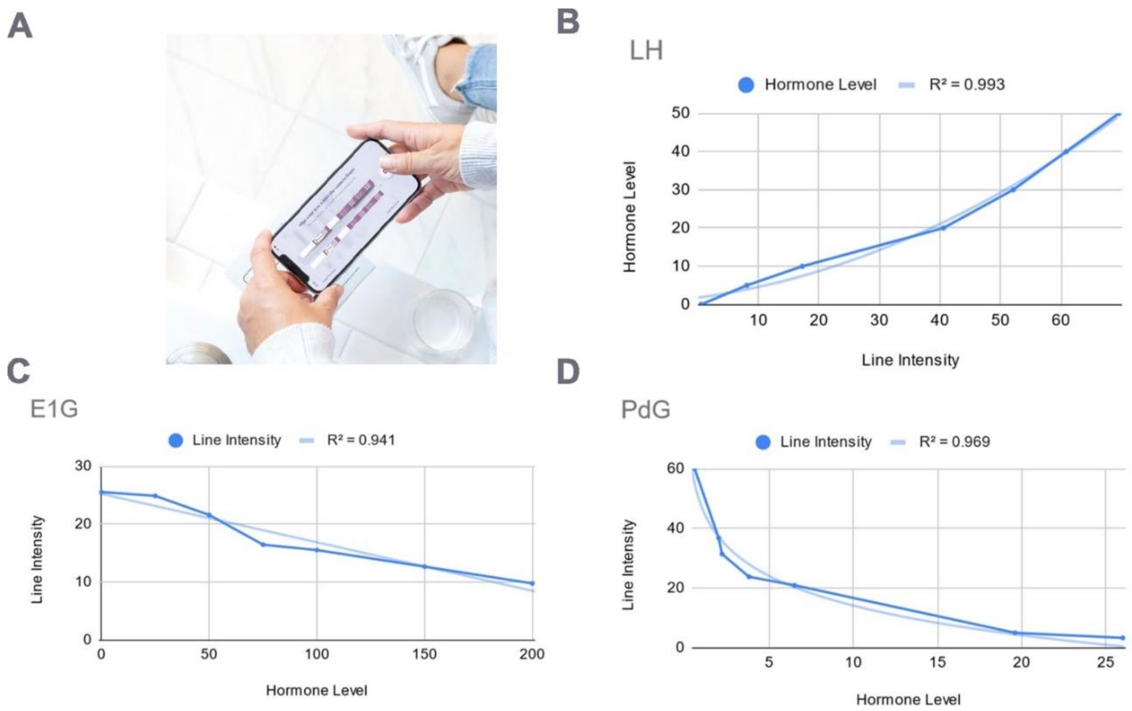

Figure S2. Proov Insight App algorithmically calculates hormone values. A) Users photograph multihormone and FSH test strips with their smartphone, guided by the Proov Insight App. The Proov Insight app uses an algorithm to calculate the hormone level based on the intensity of the line corresponding to that hormone, according to the following fits: B) 2nd-Degree Polynomial, LH C) Linear (inverse), E1G D) Logarithmic, PdG .

Table S1. Multihormone strips show no cross-reactivity to tested compounds.

Substance	Concentration	Substance	Concentration
LH	600 mIU/ml	Hemoglobin	500 mg/dl
HCG	1000 mIU/ml	Tetracycline	20 mg/dl
Progesterone	100 ng/ml	Nitrite Positive	1%
PdG	60 ug/ml	Phenothiazine	20 mg/dl
Estone-3-Glucuronide	600 ng/ml	Ethanol	1%
Acetaminophen	20 mg/dl	Albumin	100 mg/dl
Ascorbic Acid	20 mg/dl	FSH	150 mIU/ml
Caffeine	20 mg/dl	pH 4	NA
Glucose	2000 mg/dl	pH 5	NA
Ampicillin	20 mg/dl	pH 8	NA
Ketone	1%	pH 9	NA
Acetylsalicylic Acid	20 mg/dl	Specific Gravity	1.00
Atropine	20 mg/dl	Specific Gravity	1.015
Gentisic Acid	20 mg/dl	Specific Gravity	1.025

Table S2. Diagnoses and Treatments.

Diagnosis	Number of Women in Study
PCOS	3
Diminished Ovarian Reserve	4
Recurrent Pregnancy Loss	3
Endometriosis	1
Asherman Syndrome	1
**Treatment Used**	
Letrozole	6
Clomid	3
Progesterone	9
hCG Trigger	2
